# Association of adiponectin levels with polycystic ovary syndrome among Indian women

**DOI:** 10.6026/97320630018864

**Published:** 2022-10-31

**Authors:** Sudha Patil, GK Veerabhadra Goud, Rohini Nanjundappa Shivashankar, Senigala Kuruba Anusuya, Veluri Ganesh

**Affiliations:** 1 Department of Obstetrics and Gynaecology, Andaman and Nicobar Islands Institute of Medical Sciences, Port Blair - 744104, Andaman and Nicobar Islands, India; 2 Department of Biochemistry, Andaman and Nicobar Islands Institute of Medical Sciences, Port Blair - 744104,Andaman and Nicobar Islands, India; 3 Department of Obstetrics and Gynaecology, Akash Institute of Medical Sciences and Research Centre, Bangalore - 562110, Karnataka, India; 4 Department of Biochemistry, Akash Institute of Medical Sciences and Research Centre, Bangalore - 562110, Karnataka, India

**Keywords:** Adiponectin, BMI and poly cystic ovary syndrome

## Abstract

Polycystic Ovary Syndrome (PCOS) is a hormonal condition where women reproductive hormones are out of endocrinal balance. Women with PCOS frequently exhibit obesity, which causes irregularities in the levels of adipocytes like adiponectin. It is hypothesized that the altered adiponectin levels also play a role in the endocrine and metabolic abnormalities seen in PCOS women. We aimed to study the association of adiponectin levels with PCOS women. This case control study included 60 PCOS women under 30 age, and BMI matched healthy controls were recruited. For all the subjects Biochemical and serum adiponectin were determined. The serum adiponectin shows significantly decreased in PCOS women when compared to healthy controls (P <0.001**). This levels were inversely associated with BMI and total cholesterol, triglycerides in women with PCOS (P<0.001**). The significantly decreased levels of serum adiponectin can be useful for diagnostic and prognostic tool for PCOS in women and its application can be used for the success of specific emerging treatment modalities where the follow up shows improvement of levels of adiponectin.

## Background:

Polycystic Ovary Syndrome (PCOS) is a hormonal disharmony that women can face during their childbearing years. It can affect one’s ability to have a child. It can cause amenorrhea, delayed cycles or have unpredictable cycles, Acne, unwanted body and facial hair and risk of other health problems, including diabetes and high blood pressure [1]. When the patients have PCOS reproductive hormones i.e luteinizing hormone, follicle stimulating hormones and androgen levels are altered. This can lead to dysfunction with ovaries, affecting menstrual cycle length and fertility [2]. The women have too high insulin and also blood sugars and the patient with PCOS body might not react to insulin the way it should, and low progesterone in PCOS patients. The low levels of this hormone results in missed periods for a long time or have trouble predicting when they'll come [3]. The classical symptoms of PCOS are missed, irregular, infrequent, or prolonged periods. Excess androgens can cause hair loss hair in places don’t want it and acne. Other symptoms include darkened skin or excess skin on the neck or in the armpits mood changes pelvic pain weight gain around your middle [4]. Although PCOS is thought to be the most prevalent endocrinopathy affecting women of childbearing age, estimates of its prevalence range greatly from 2.2% to 26%. The disparity in the diagnostic criteria is one of the factors contributing to the prevalence reports' wide range. These include the Rotterdam criteria, the Androgen Excess Society (AES) criteria, and the National Institute of Health (NIH) criteria [5]. Another recent study of the prevalence of PCOS in a large birth cohort of 728 women born in a single maternity hospital in Australia between 1973 and 1975 found that the prevalence was estimated to be 8.7 2.0% using NIH criteria, 11.9 2.4% when using Rotterdam criteria, and 10.2 2.2% when using AES criteria. High incidence of PCOS has been recorded, estimated to be around 9.13% in teenagers, even in Indian women [6]. According to Another recent study community-based cross-sectional study of 778 young and adolescent girls between the ages of 15 and 24, the prevalence of PCOS is 10.7% according to the Androgen Excess Society criteria and 22.5% according to the Rotterdam criteria [7]. According to a study from South India that involved 126 teenage girls between the ages of 12 and 19 years, urban girls were more likely than girls from rural regions to have PCOS. These studies' findings suggest that PCOS manifests itself early in the reproductive age and calls for early detection [8]. Despite being the most prevalent endocrine condition in women and having been known for a long time, PCOS' pathogenesis is still poorly understood and is thought to involve multiple factors [9]. According to some theories, the disease results from a complicated interaction between intrinsic individual traits and environmental influences. These interactions cause hormonal imbalances that result in a condition of hyper androgenemia, which is a defining feature of PCOS and also causes its clinical symptoms [10]. The emergence of PCOS is significantly influenced by genetic factors. With varied penetrance influenced by a variety of environmental and epigenetic factors, the disease is inherited in an autosomal dominant pattern of mendelian heredity. The ovarian and/or adrenal systems are the source of the hyper androgenism. Genetic and molecular investigations have shown that theca cells from polycystic ovaries have an innate steroidogenic abnormality that leads to an increase in androgen production [11]. This in turn entails increased activity of three enzymes, namely 17-α hydroxyls, 3-β hydroxyl steroid Dehydrogenase and side chain cleavage enzyme that act in the steroidogenic pathway. An essential protein produced and secreted by adipose tissue is called adiponectin. Historically, adipose tissue has been thought of as an inert organ that stores fat. Adipose tissue, however, is increasingly recognized as a metabolically active organ, contributing to a variety of metabolic processes by releasing a number of locally and systemically physiologically active chemicals known as adipocytes. Adiponectin also has additional effects through several mechanisms, including those that are anti-inflammatory, antioxidant, vasoprotective, cardio protective, renoprotective, anti-apoptotic, and atheroprotective [12]. Adipocytokines, in particular adiponectin, have recently been linked to the pathogenesis of PCOS. Adiponectin levels were discovered to be inversely correlated with obesity in healthy people, and they were also found to be regulated by the degree of insulin resistance and hyper insulinemia. According to studies, women with PCOS have reduced amounts of circulating adiponectin, which adds to the problems related to PCOS. The altered levels may develop spontaneously or as a result of the increased obesity or insulin resistance that are frequently seen in these women of these factors. The decreased adiponectin levels may further cause decreased insulin sensitivity leading to insulin resistance, thus resulting in the development of a vicious circle [13]. Women with PCOS frequently exhibit obesity, which causes irregularities in the levels of adipocytes like adiponectin. It is hypothesized that the altered adiponectin levels also play a role in the endocrine and metabolic abnormalities seen in PCOS women. The altered adiponectin levels do, however, represent a critical link between PCOS and the related endocrine and metabolic problems. Weight loss and treatment interventions may affect adiponectin levels. The pathophysiology of PCOS and its effects may be better understood by measuring the adiponectin levels in PCOS patients. These have clinical significance for adiponectin-focused treatment strategies that aim to reduce polycystic ovarian syndrome-related consequences [14]. Some of the studies reported serum adiponectin was significantly elevated in PCOS women and another studies found serum adiponectin levels in PCOS women. Inconsistent results of serum adiponectin in PCOS women, based on this background the present study aimed to evaluate the serum adiponectin concentrations in women with poly cystic ovary syndrome.

## Material and Methods:

The present cross sectional study included a total of sixty (60) women attending to Obstetrics and Gynaecology OPD, at Akash Institute of Medical Science and Research Center, Devanahalli Bangalore Rural, Karnataka and diagnosed with polycystic ovary syndrome based on Rotterdam criteria. Thirty (30) age and BMI matched healthy women were recruited as controls. All the participants were included after an informed consent. The study was approved by the institutional ethics committee (IEC No: 316/2015-16).

### Criteria of study:

#### Inclusion criteria:

Women of reproductive age diagnosed with PCOS as per Rotterdam criteria (Clinical and biochemical hyper androgenism, oligo ovulation/an ovulation and Polycystic ovaries).

#### Exclusion criteria:

Women with history of smoking, alcoholism, diabetes, hypertension, renal diseases, liver diseases, thyroid diseases, cardiovascular diseases (CVD), acute infections and Congenital adrenal hyperplasia non classic adrenal hyperplasia Cushing syndrome Androgen secreting tumors Idiopathic hyper androgenism Idiopathic hirsutism Hyper prolactinemia. People on treatment such as hormone therapy, metformin, thiazolidinediones, oral contraceptives, and steroids were excluded from this study.

### Sample collection:

Five (5) mL of fasting venous blood sample was collected from all the subjects into two tubes: 1mL into a tube containing anticoagulant, and 4 mL into a plain tube. The collected blood samples were separated immediately and plain samples were allowed to clot and separated by centrifugation at 3000 rpm for 10 min. The separated plasma and serum samples were transferred into appropriately labeled aliquots and stored at -80°C until biochemical analysis was done.

### Methods:

Fasting and post parandial blood sugars levels were measured by using the glucose oxidase peroxidase (GOD-POD) method, total cholesterol was estimated by using cholesterol oxidase and peroxidase method (COD-POD) method and triglycerides was analysed by enzymatic method, high density lipoprotein (HDL) cholesterol was estimated by selective inhibitory method. All the biochemical parameters analyzed by using fully automatic analyzer (erba EM -200). Serum adiponectin were determined by using Enzyme Linked Immuno Sorbent Assay (ELISA, Euro Immune Fully Automatic Analyzer).

### Statistical analysis:

The Kolmogorov-Smirnov test was used to determine whether the data were normal. For normally and non-normally distributed data, the data were reported as mean standard deviation or median (inter quartile range), respectively. Analysis of variance (ANOVA) used for comparison of parameters among the groups was completed. The Pearson or Spearman rank correlation was used to investigate the Association between the variables. With the help of SPSS for Windows version 16.0 and Microsoft Excel spreadsheets, statistical analysis was carried out. Statistical significance was defined as a p value 0.05.

## Results:

Table 1(see PDF) lists the demographic details and biochemical measurements examined in PCOS and healthy control women. Age-wise, both groups were matched, and obese PCOS women outperformed healthy women (p <0.001**). Total cholesterol and triglycerides in PCOS women were considerably higher than in controls (p = 0.001** and 0.014* for total cholesterol and triglycerides, respectively). When compared to healthy controls, PCOS women's serum adiponectin levels was shown to be considerably significantly lower (p=0.001**). Table 2(see PDF) shows the relationships between adiponectin and the factors investigated using Pearson or Spearman rank correlation analysis. The adiponectin was positively correlated with FBS, TGL and negatively correlated with age, BMI, TC, HDL-C. No significant correlation between adiponectin and any of the factors was found. Figure 1(see PDF) illustrates the serum adiponectin concentrations between PCOS women and healthy controls. The women with PCOS shown significantly decreased concentrations of serum adiponectin when compared to healthy controls (P = 0.001**). Figure 2(see PDF) shows the pearson’s correlation analysis between serum adiponectin and age, BMI among study subjects. There was a negative correlation between the serum adiponectin and age (r = -0.347). The serum adiponectin also negatively correlated with BMI (r=-0.207). Figure 3(see PDF) shows the correlation between biochemical parameters and adiponectin among the study subjects. The serum adiponectin negatively correlated with total cholesterol, HDL (r = -0.719 and -0.017) and there was no correlation between serum adiponectin and FBS, TGL (r = 0.210 and 0.093).

## Discussion:

One of the most prevalent endocrine-metabolic disorders affecting women of reproductive age is polycystic ovarian syndrome (PCOS). PCOS develops early in the reproductive age as a result of interactions between innate and environmental variables. In the current study, polycystic ovary syndrome in women was identified using Rotterdam criteria [15]. Along with hormonal abnormalities, polycystic ovarian syndrome frequently includes metabolic issues as dyslipidemia and glucose intolerance. Obesity, which is present in more than 50% of PCOS-affected women, is a major contributor to the metabolic side effects of PCOS. Women with polycystic ovarian syndrome had significantly lower serum adiponectin levels than healthy females in the current study (10.19 ± 6.11 Vs 7.16±3.28, p <0.001**) (Table 1- see PDF). Numerous researches have assessed the connection between PCOS and adiponectin, too. Most studies show that PCOS women's adiponectin levels are considerably lower than those of BMI-matched healthy controls [16]. In the current study, PCOS women were overweight and had a BMI that was considerably higher than controls (24.68 ± 6.00 Vs. 28.68 ± 7.52, p<0.001**) (Table 1 - see PDF). There have been several theories put up as to why PCOS women have decreased amounts of adiponectin. While some studies have shown that insulin resistance and glucose intolerance are to blame for the changes in adiponectin levels, others have demonstrated that adiponectin concentration varies with the degree of obesity and is unaffected by insulin resistance [17]. Hypo adiponectinaemia has been found in both obese and lean women with PCOS who exhibit varying degrees of insulin resistance. However, another recent study who also found decreased levels of total adiponectin and HMW adiponectin in PCOS women hypothesized that the decreased levels of adiponectin occur independently of BMI and insulin resistance and that posttranscriptional/translational modifications are responsible for the low levels of HMW adiponectin in PCOS. When compared to weight-matched women without PCOS, individuals with PCOS exhibit considerably lower messenger RNA (mRNA) expression for adiponectin in their adipose tissue, according to research [18]. The reduced levels of circulating adiponectin that are detected in women with PCOS were shown to be consistent with the decreased expression of adiponectin mRNA, which was observed in both subcutaneous and visceral fat tissue. The increased adiposity, as shown by a higher BMI in women with polycystic ovarian syndrome in the present study, may be the cause of the decreased adiponectin levels seen in them, according to an examination of adipose tissue in women with PCOS. However, Arikan et al. found higher levels of adiponectin in young, non-obese PCOS patients compared to controls [19]. As a result of its function in lipid and carbohydrate metabolism, adiponectin actively contributes to energy homeostasis. It has been demonstrated that adiponectin controls the triglyceride-rich lipoproteins and the enzymes responsible for controlling lipid metabolism. There have been reports of altered adiponectin levels in connection to lipid disruptions in women with PCOS. Serum lipid profiles were assessed in the current investigation and compared between PCOS women and healthy controls. In contrast to controls, PCOS women exhibited similar levels of HDL cholesterol (p=0.116), but considerably higher total cholesterol and triglyceride levels (p=0.014* and 0.001** for total cholesterol and triglycerides, respectively) (Table 1 - see PDF). Women with PCOS frequently experience dyslipidemia, which raises their risk of cardiovascular disease and the metabolic syndrome [20]. Previous research found that PCOS women had lower HDL cholesterol and higher total and triglycerides when compared to controls. Multiple factors can lead to dyslipidemia in the context of polycystic ovarian syndrome. The lipoprotein abnormalities seen in PCOS women have all been theorized to be caused by the higher prevalence of obesity, insulin resistance, and hyper androgenemia. In the presence of insulin resistance, increased triglyceride levels are caused by increased lipogenesis, decreased clearance, reduced fatty acid oxidation and their increased availability, as well as an increased secretion of very low density lipoprotein (VLDL) particles by the hepatocytes [21]. With the use of Pearson correlation and Spearman rank correlation analysis, the relationship between adiponectin and the clinical and biochemical markers was examined. Age and BMI were negatively but not significantly correlated with adiponectin (Table 1 - see PDF). While another recent study found that adiponectin was linked to obesity in their study of sixty women with PCOS, Another recent study found a strong negative association between adiponectin and age in PCOS women [22].Therefore, the results of this study show that blood adiponectin levels are considerably lower in polycystic ovarian syndrome patients than in healthy controls. One of the defining characteristics of PCOS is hyper androgenemia, which causes obesity and lower levels of adiponectin as a result. Low adiponectin levels can contribute to PCOS's metabolic side effects, such as insulin resistance and dyslipidemia. Therefore, a significant relationship between obesity and the side effects of PCOS appears to be formed by the altered adiponectin levels in PCOS [23]. Adiponectin levels are known to rise as a result of therapeutic procedures involving medications like metformin and weight loss programmers, which may have positive consequences [24]. Therefore, although adiponectin has been linked to the hormonal and metabolic abnormalities of PCOS, more research is needed to determine its precise function in polycystic ovarian syndrome (PCOS) patients.

## Conclusions:

Serum adiponectin can be useful for diagnostic and prognostic tool for PCOS in women and its application can be used for the success of specific emerging treatment modalities where the follow up shows improvement of levels of adiponectin.

## Limitations:

In our study less sample size and follow up is not there, furthermore there is a need large sample size and follow up studies are required for serum adiponectin evaluation in PCOS women.
